# Homogeneous luminescent quantitation of cellular guanosine and adenosine triphosphates (GTP and ATP) using QT-Luc^GTP&ATP^ assay

**DOI:** 10.1007/s00216-023-04944-9

**Published:** 2023-09-16

**Authors:** Kari Kopra, Randa Mahran, Titta Yli-Hollo, Sho Tabata, Emmiliisa Vuorinen, Yuki Fujii, Iida Vuorinen, Aki Ogawa-Iio, Akiyoshi Hirayama, Tomoyoshi Soga, Atsuo T. Sasaki, Harri Härmä

**Affiliations:** 1 Department of Chemistry, University of Turku, Henrikinkatu 2, 20500 Turku, Finland; 2 Institute for Advanced Biosciences, Keio University, Tsuruoka, Yamagata 997-0052 Japan; 3 Department of Internal Medicine, University of Cincinnati College of Medicine, 3125 Eden Ave, Cincinnati, OH 45267-0508 USA; 4 Department of Clinical and Molecular Genetics, Hiroshima University Hospital, Hiroshima, 734-8551 Japan

**Keywords:** Adenosine triphosphate (ATP), Capillary electrophoresis (CE), Guanosine triphosphate (GTP), Immunoassay, Mass spectrometry (MS), Time-resolved luminescence (TRL)

## Abstract

Guanosine triphosphate (GTP) and adenosine triphosphate (ATP) are essential nucleic acid building blocks and serve as energy molecules for a wide range of cellular reactions. Cellular GTP concentration fluctuates independently of ATP and is significantly elevated in numerous cancers, contributing to malignancy. Quantitative measurement of ATP and GTP has become increasingly important to elucidate how concentration changes regulate cell function. Liquid chromatography–coupled mass spectrometry (LC–MS) and capillary electrophoresis-coupled MS (CE–MS) are powerful methods widely used for the identification and quantification of biological metabolites. However, these methods have limitations related to specialized instrumentation and expertise, low throughput, and high costs. Here, we introduce a novel quantitative method for GTP concentration monitoring (GTP-quenching resonance energy transfer (QRET)) in homogenous cellular extracts. CE–MS analysis along with pharmacological control of cellular GTP levels shows that GTP-QRET possesses high dynamic range and accuracy. Furthermore, we combined GTP-QRET with luciferase-based ATP detection, leading to a new technology, termed QT-Luc^GTP&ATP^, enabling high-throughput compatible dual monitoring of cellular GTP and ATP in a homogenous fashion. Collectively, GTP-QRET and QT-Luc^GTP&ATP^ offer a unique, high-throughput opportunity to explore cellular energy metabolism, serving as a powerful platform for the development of novel therapeutics and extending its usability across a range of disciplines.

**Graphical Abstract:**

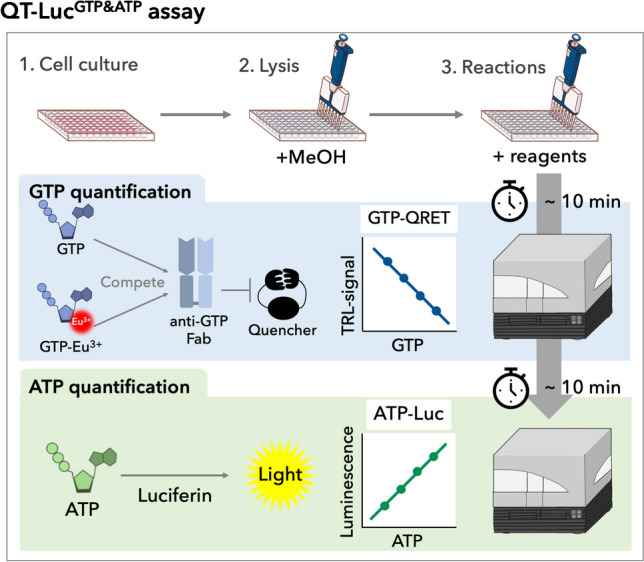

**Supplementary Information:**

The online version contains supplementary material available at 10.1007/s00216-023-04944-9.

## Introduction

Nucleotides are organic molecules composed of pentose sugar, phosphate ester, and varying nitrogenous base moieties (see Electronic Supplementary Material Fig. S1). Apart from serving as building blocks for RNA synthesis, the triphosphate forms of adenine and guanine nucleotides, ATP and GTP, function as energy molecules that drive a multitude of cellular processes. However, their roles exhibit a clear distinction [1, 2]. ATP is involved in nearly all cellular metabolic reactions and is necessary also for GTP synthesis [3, 4]. In mammalian cells, cellular ATP concentrations are consistently high, typically maintained at levels of 1–5 mM [5]. In contrast, GTP primarily drives protein synthesis and regulation, cytoskeleton organization, membrane transport, and signal transduction. The cellular GTP concentrations are more variable than those for ATP, fluctuating between 0.1 and 1 mM. GTP levels are regulated differently depending on tissue and cell type, and the GTP-to-ATP ratio varies significantly [5, 6]. GTP concentration is generally increased in cells undergoing proliferation, as documented by a wide range of organisms [6, 7].

Dysregulation of GTP synthesis has been linked to several inherited diseases. The range of phenotypes highlights the importance of GTP metabolism regulation in vivo for brain function, vision, and the immune system. For instance, deletion mutations in HPRT1 (hypoxanthine-phosphoribosyltransferase-1) can cause hyperuricemia, resulting in severe gout and acute renal failure (Kelley–Seegmiller syndrome) [8–10]. Also, a substantial loss of HPRT1 activity contributes to self-injurious behaviors and motor and cognitive dysfunction (Lesch–Nyhan syndrome) [11–13]. The retina has particularly high GTP concentrations [5, 14], and it relies on photoreceptor cells in converting GTP to cGMP upon light stimulation, triggering input signals to the optic nerve. Loss-of-function mutations in IMPDH1 (inosine monophosphate dehydrogenase 1), the rate-limiting enzyme for de novo GTP synthesis [15], cause retinitis pigmentosa. IMPDH1 dysfunction results in retinal degeneration, accelerated visual aging, and blindness [16–18]. IMPDH inhibitors, including mycophenolic acid (MPA), exhibit immunosuppressive effects and are employed to suppress rejection in organ transplantation and treat autoimmune diseases like systemic lupus erythematosus (SLE). This indicates GTP’s vital role in immune system operation [15, 19]. GTP metabolic reprogramming occurs in cancer cells, where high GTP concentrations promote cellular anabolism, potentially making the GTP metabolic system a target for cancer therapy. However, despite considerable research, our understanding of the comprehensive and precise roles of nucleotides under physiological and pathological conditions remains limited. Furthermore, the implications of alterations in nucleotide levels in relation to various diseases have yet to be fully elucidated. While the mechanism translating changes in GTP concentrations into cellular and biological functions remains elusive, evidently, it is crucial to monitor cellular GTP levels and define its ratio against ATP. This would give us a better understanding on the role of cellular energy metabolism and might enable development of novel therapeutics for diseases associated with dysregulated GTP metabolism [20].

The biological relevance of nucleotides and their involvement in various diseases underscores the necessity for a straightforward and precise intracellular-nucleotide-level monitoring tool. Numerous methods have been developed to specifically measure adenosine- and guanosine-related nucleotides using high-performance liquid chromatography (HPLC) coupled with ultraviolet–visible light detection [21–23]. More recent approaches employ mass spectrometry (MS) as a highly selective and sensitive detection method. In these techniques, quantification has been performed across various matrices and for multiple nucleotides [24–28]. However, most of these methods rely on direct analysis, in which phosphate fractions are separated on an HPLC column using ion-pairing-based mobile phases. This separation approach may induce ion suppression from the mobile phase and may consequently impact the measurement of other molecules using HPLC. To address these limitations, a capillary electrophoresis–MS (CE-MS) method has been developed [29–31]. CE is first used to separate metabolites based on their charge and size, followed by selective detection through MS. The major advantages of CE–MS include its high resolution and the capability to analyze nearly any charged species, encompassing both cationic and anionic analytes [32–34]. These methods possess significant advantages in terms of accuracy, specificity, sensitivity, and dynamic range. However, all separation-based techniques require expertise and specialized equipment, such as HPLC and MS apparatus. As a result, only a limited number of researchers can perform these assays. Furthermore, these methods generally require relatively large amounts of biological samples, typically over 10,000 cells for each run, which limits the use of multi-well plate cultures and increases costs. Consequently, it has been challenging to perform high-throughput analysis for GTP and ATP concentrations, which hampers our understanding of their precise roles in cellular functions, disease progression, and drug screenings. Currently, there are high-throughput screening methods available enabling luminescence-based ATP detection, but the same is not true for GTP [35–37]. In addition to luminescence, there have been several attempts to develop fluorescent probes for live cell monitoring of ATP and GTP, but none of these methods for GTP has reached higher popularity, because of the complexity, low sensitivity and selectivity, and the need for specialized expertise and equipment [38]. However, especially aptamer-based detection strategies have already shown promises for also intracellular nucleotide monitoring [39–41].

In our previous work, we identified GTP-specific single-chain variable fragments (scFv) from the synthetic antibody fragment library by phage display screening, and converted that to the first GTP-specific antigen-binding fragment (Fab) [42, 43]. The anti-GTP Fab clone 2A4 (hereafter 2A4^GTP^ Fab) has shown superior specificity to GTP over GDP and ATP in vitro, which we have utilized to measure the rate of GTP hydrolysis activity by 2A4^GTP^ Fab and Eu^3+^-GTP in a homogenous assay format. The principle underlying the monitoring of GTP consumption is based on the 2A4^GTP^ Fab competition between GTP and Eu^3+^-GTP, which is detected using the single-label quenching resonance energy transfer (QRET) principle and time-resolved luminescence (TRL) readout [42]. While the 2A4^GTP^ Fab-based GTP detection with QRET enables nanomolar sensitivity using pure solutions and proteins, it has remained untested whether it can still selectively react with GTP in the presence of cellular extracts that contain thousands of metabolites and proteins. Cellular extracts have a high ATP concentration, typically five- to tenfold higher than that of GTP [5]. Likewise, cellular concentrations of the other nucleotides (e.g., UTP, CTP) and nucleotide derivatives (e.g., nicotinamide adenine dinucleotide, NAD^+^, and S-adenosyl methionine, SAM) have comparable ranges to GTP [44, 45]. Thus, 2A4^GTP^ Fab may cross-react with many of these metabolites in the presence of homogenous cellular extract or possess a polyvalent feature binding some other cellular components [46]. To answer these concerns, here, we studied a series of key parameters of the GTP-QRET system and conducted a rigorous validation using CE-MS as a reference method. We were able to verify the functionality of 2A4^GTP^ Fab under homogenous conditions. Moreover, we successfully developed a QT-Luc^GTP&ATP^ platform to monitor both GTP and ATP from the same sample in the same well by combining the GTP-QRET platform with direct luminescence–based ATP monitoring. This technique, termed QT-Luc^GTP&ATP^, can be applied to 6- to 384-well-plate formats, and results can be obtained in less than an hour with as few as 100 cells/well. Given the current extensive applicability of ATP detection, the simultaneous detection of GTP and ATP in a multi-well plate format presents significant potential for establishing a new QT-Luc^GTP&ATP^ cost-effective technological platform with broad application across numerous fields, encompassing medicine, pharmacology, agriculture, and life, food, and analytical sciences.

## Experimental section

### Materials and apparatus

Nonadentate europium-chelate-9d, {2,2′,2″,2′″-{[4′-(4′″-isothiocyanatophenyl)-2,2′,6′,2″-terpyridine-6,6″-diyl]bis(methylene-nitrilo)}tetrakis(acetate)}europium(III), used for Eu^3+^-GTP conjugation, and the soluble quencher molecule, named MT2, were obtained from QRET Technologies (Turku, Finland). Labels were used according to the manufacturer’s instructions, and purification and concentration determination was performed as previously described [47–49]. A ReadiUse™ Rapid Luminometric ATP Assay Kit was obtained from AAT Bioquest. White Corning 384-well low-volume assay plates were used in all GTP-QRET and QT-Luc^GTP&ATP^ assays for the detection of GTP and ATP. The 96-well Costar tissue culture plates (Corning, NY, USA) and black 384-well Optiplates (PerkinElmer, Netherlands) were used in cell sample preparation. Cell lines (U87MG, HEK293T, A549, MDA-MB-468, QGP1, BT-474, HeLa, and HTC116) were obtained from the American Type Culture Collection (ATCC, Manassas, VA, USA), and all larger cultures were performed in a 6-well plate or a T75 culture flask (Corning). Dulbecco’s modified Eagle medium (DMEM), Roswell Park Memorial Institute (RPMI) 1640 medium, fetal bovine serum, trypsin/EDTA, l-glutamine, and penicillin/streptomycin were purchased from (Gibco, Thermo Fisher Scientific, Waltham, MA, USA). Phosphate-buffered saline (PBS) without calcium and magnesium was from Lonza (Walkersville, USA). Normocin was from InvivoGen (USA). All nucleotide phosphates, GTP, ATP, guanosine-5′-diphosphate (GDP), guanosine-5′-monophosphate (GMP), cytidine-5′-triphosphate (CTP), and uridine-5′-triphosphate (UTP) were from Jena Bioscience (Jena, Germany) and Sigma-Aldrich (St. Louis, MO, USA). All other reagents, including analytical-grade solvents, buffer components, guanosine, mannitol, methionine sulfone, ethane sulfonic acid, d-camphor-10-sulfonic acid, chloroform, 1,3,5-benzene tricarboxylic acid, 3-aminopyrrolidine, and MPA were from Sigma-Aldrich.

A reverse-phase liquid chromatography Dionex ultimate 3000 LC system (Dionex Corporation, Sunnyvale, CA, USA) and an Ascentis RP-amide C18 column (Sigma-Aldrich, Supelco Analytical) were used for Eu^3+^-GTP purification [47–49]. All measurements were performed using a Spark 20 M from Tecan Life Sciences (Männedorf, Switzerland). Time-resolved luminescence (TRL) measurements for GTP were performed at 620 nm, using a 340-nm excitation wavelength (800 µs delay and 400 µs decay). Total luminescence for ATP measurement was monitored using 1000 ms of integration time. CE-MS analysis was performed using an Agilent G7100 CE system (Santa Clara, CA, USA), with an Agilent 6210 time-of-flight mass spectrometer (TOFMS), Agilent1200 series isocratic HPLC pump, and Agilent G1607A CE-ESI–MS sprayer kit. For ATP and GTP analysis, the original Agilent SST316Ti stainless steel ESI needle was replaced with platinum [50].

### Cell culturing and sample preparation

The cell lines used were all cultured in a humidified atmosphere of 5% CO_2_ at 37 °C. Culturing of U87MG, HEK293T, A549, MDA-MB-468, HeLa, and HTC116 was performed in DMEM and QGP1 and BT-474 in RPMI 1640 media supplemented with 10% fetal bovine serum (FBS), 1% penicillin/streptomycin, and 2 mM l-glutamine.

For comparison of QT-Luc^GTP&ATP^ and CE-MS, subconfluent cells in a 6-well plate were washed with 5% (w/v) mannitol and dissolved in 2 mL of methanol (MeOH) containing internal standards for CE-MS (25 μM each of methionine sulfone, ethane sulfonic acid, and d-camphor-10-sulfonic acid). This solution was directly used for QT-Luc^GTP&ATP^ or dried and used after reconstitution. For CE-MS, 400 μL of this homogenate, 200 μL of Milli-Q water, and 400 μL of chloroform were mixed. After centrifugation (12,000 g for 15 min at 4 °C), the separated upper aqueous layer was filtered through a Millipore 5-kDa cutoff filter (Millipore, Bedford, MA, USA) to exclude proteins. The filtrate was freeze-dried and resolved in 25 µL of Milli-Q water containing internal standards (200 µM each of 1,3,5-benzene tricarboxylic acid and 3-aminopyrrolidine) prior to the analysis using CE-MS.

For the 96-well plate test, 10,000 cells (U87MG and HEK293T) were transferred to each well in a 96-well plate and cultured to 60–80% confluence. For MPA (1–100 µM) and guanosine (100 µM) testing, cells were further treated for 4 h by adding these compounds in a fresh media. Thereafter, the medium was aspirated, and cells were washed with PBS before 80% MeOH was added (100 µL). Plates were centrifuged at 1600 g for 10 min at room temperature (RT), and 2 µL of MeOH solution containing the nucleotide extracts was directly used for GTP-QRET or QT-Luc^GTP&ATP^ in a 384-well plate (100–1000 cells/well).

### Luminescence-based GTP and ATP monitoring

GTP-QRET assay optimization was performed using pure GTP and ATP samples (0–10 µM) and detection components in varying concentrations, Eu^3+^-GTP (2–20 nM), anti-GTP 2A4^GTP^ Fab (5–50 nM), and MT2 modulator (1.5–5 µM). Tests were performed in a preselected GTP buffer (25 mM HEPES, pH 7.5, 1 mM MgCl_2_, 0.01% Triton X-100). All assays were performed in 10 µL final volume in a white 384-well plate. ATP assay was separately optimized in the same plate using 10–25 µL final volumes. Nucleotides were in all cases added in 5 µL and ATP detection in 5–20 µL volume. In both assays, TRL- and luminescence signals were monitored at multiple time points between 5 and 60 min. Optimized conditions for each assay were used for specificity analysis and assayed separately. Titrated (0–500 µM) nucleotides (GTP, GDP, GMP, ATP, UTP, and CTP) were added in 5 µL and pre-made and optimized detection solution, Eu^3+^-GTP (10 nM), anti-GTP 2A4^GTP^ Fab (20 nM), and MT2 quencher (2.7 µM), in 5 µL. Concentrations are reported in final 10 µL volume. Nucleotides were similarly titrated with the ReadiUse™ Rapid Luminometric ATP Assay Kit using 10-µL nucleotide samples and 10 µL of the ATP detection reagent in a total of 20-µL final volumes. In both assays, signals were monitored after 15 min of incubation at RT.

Two individual assays were combined by testing nucleotide (GTP, ATP, and ATP + GTP) addition in Milli-Q water, GTP buffer, and GTP buffer supplemented with MeOH (2–28%). In all assays, the nucleotide sample was added in 5 µL followed by GTP detection solution addition (5 µL), and TRL-signal detection (15 min). ATP detection solution was added in 10 µL on top of GTP assay components, and the luminescence signal was monitored (15 min). Nucleotides extracted from the cells were measured using the same protocol, with the exception that standard buffer’s MeOH concentration was adjusted to match with the cell samples. Typically, MeOH concentration was below 8%. Cells cultured in a 96-well plate were monitored similarly to other samples except that nucleotides were added in 2 µL and GTP detection in 8 µL. GTP detection solution concentrations in the final 10-µL volume were the same in all assays.

### Quantifications of ATP and GTP using CE-TOFMS

Cellular ATP and GTP were detected and quantified using time-of-flight CE–TOFMS (Agilent Technologies) as previously reported [31, 51, 52]. The raw data were processed with MasterHands [53]. ATP and GTP were identified by matching their m/z values and migration times to standard compounds. A series of other metabolites were analyzed in a similar manner.

### Data analysis

In all assays, the signal-to-background ratio (S/B) was calculated as *µ*_max_/*µ*_min_ either by using the true minimum and maximum or from the linear range of the assay. The coefficient of variation (CV%) was calculated as (*σ*/*µ*)*100. In both formulas, *µ* is the mean value and *σ* is the standard deviation (SD). Data were analyzed using Origin 8 software (OriginLab, Northampton, MA) and basic linear and sigmoidal fitting functions. The half-maximal effective concentration (EC_50_) values were obtained from sigmoidal fitting, and ATP and GTP concentrations were determined based on the linear part of the appropriate standards.

## Results and discussion

### 2A4^GTP^ Fab preferentially binds GTP over GDP, GMP, ATP, CTP, and UTP

In the previous study, we monitored GTP consumption in vitro with the 2A4^GTP^, using purified recombinant GTPases [42, 43]. While 2A4^GTP^ Fab displayed remarkable sensitivity and specificity toward GTP over GDP in vitro, whether 2A4^GTP^ Fab can discriminate GTP from cellular extracts remains untested. Given that a cell contains thousands of metabolites and proteins, it is possible that these cellular components may interfere with the reactivity of 2A4^GTP^ Fab toward GTP. Nucleotide structures are highly similar, and thus, for the 2A4^GTP^ Fab, it is important to recognize not only the triphosphate ester, but also the varying nucleotide base (see Electronic Supplementary Material Fig. S1).

Thus, we set out to test 2A4^GTP^ Fab for its guanine base recognition by comparing the reactivity to three other major ribonucleotides, ATP, UTP, and CTP. On the other hand, we compared the detectability of GTP over GDP and GMP to estimate phosphate recognition. Based on the initial tests, 10 nM Eu^3+^-GTP and 20 nM 2A4^GTP^ Fab were selected for these titrations to enable better tolerability of the cellular extracts without significantly sacrificing the sensitivity and selectivity. Under these conditions, 2A4^GTP^ Fab detects GTP 86-, 90-, and over 300-fold better than CTP, UTP, and ATP, respectively (see Electronic Supplementary Material Fig. S2). Similarly, GTP was detected at a 33-fold lower concentration than GDP and the difference was over 1300-fold with GMP (see Electronic Supplementary Material Fig. S2). Even though cellular ATP levels are typically 5- to 10-folds higher than GTP, the results suggest that 2A4^GTP^ Fab is highly likely to distinguish GTP signal from ATP in a cellular extract. Likewise, since GTP levels are typically comparable or even higher than those of CTP and UTP, and significantly higher in comparison to that of GDP (10- to 50-fold) and GMP, 2A4^GTP^ Fab is expected to detect GTP specifically in cellular extracts [5]. As the ultimate goal of the present study is to develop 2A4^GTP^ Fab-based GTP detection using cellular extract, we also tested the effect of MeOH used in nucleotide extract preparation. This method is widely used for metabolomics analysis, including CE-MS for assessing polar metabolites [53–56]. While MeOH extraction for metabolomics is typically followed by evaporation and reconstitution with water or solvent, to increase simplicity and throughput, we omitted this step, and instead, used diluted cellular extract. As shown in Fig. S3 (see Electronic Supplementary Material), 2A4^GTP^ Fab can detect GTP up to 14% MeOH without a major difference in dynamic range.

### GTP-QRET by 2A4^GTP^ Fab detects exogenously added GTP in the presence of cellular extracts

**Fig. 1:**
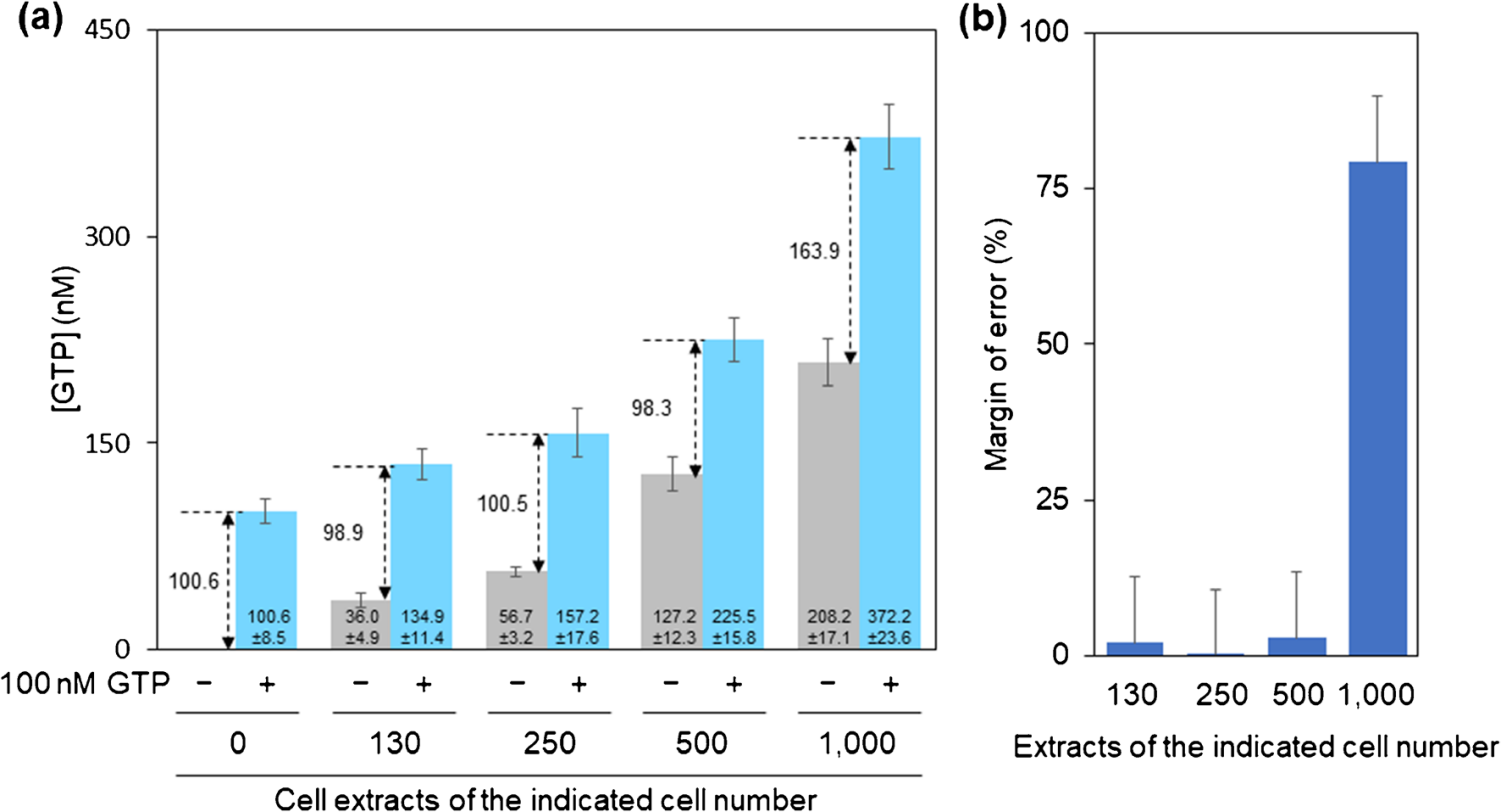
The effect of the cellular extract on the measurement of GTP concentration. **a** GTP concentration was measured in the presence or absence of diluted cellular extracts, and in the presence or absence of 100 nM GTP used as a standard. The cellular extract of U87MG cells was prepared in 80% MeOH and diluted to GTP buffer to obtain cell contents ranging from 100 to 1000 cells/well^384^. Data represent mean ± SD (*n* = 3). **b** The perturbation effect of cellular extracts on GTP-QRET is shown as a margin of error based on the result of **a**

Cellular extracts contain thousands of metabolites, which may interfere with the 2A4^GTP^ Fab recognition of GTP. To test this possibility, we measured exogenously added GTP in the presence and absence of serially diluted cellular extracts to evaluate the effect of these extracts on GTP concentration measurements. Throughout the study, we employed both 96-well and 384-well microplates for our experiments. To clearly delineate the cell number in a well of these two plates, we will henceforth utilize the notations “well^96^” and “well^384^” to represent a single well within the respective 96-well and 384-well microplates. We performed the assay using typical cell numbers in 96-well plates, simultaneously studying the high-throughput compatibility of our assay. Widely used glioblastoma U87MG cells were studied to design an experiment under culture conditions in a 96-well plate. U87MG cells proliferate in 96-well plates to a density of approximately 10,000 to 50,000 cells/well^96^, and metabolites can be extracted from the 96-well plates using 100 µL of 80% MeOH. A threefold dilution was performed to simplify the pipetting, and 2 µL of this diluted sample was subsequently analyzed in a 384-well plate using the optimized GTP-QRET. The amount of metabolite in the assay solution was estimated to be around 1.2% of the total, equating to extracts of approximately 100 cells in a well^384^. The results demonstrated that the accuracy of the GTP-QRET assay was well preserved for cell contents ranging from 100 to 500 cells/well^384^, corresponding to typical cell numbers from 12,000 to 60,000 cells/well^96^ (Fig. 1). In contrast, when cellular extracts reached 1000 cells/well^384^ (roughly 120,000 cells/well^96^), which are uncommon densities used for the biological assay, the GTP concentration measured by GTP-QRET appeared higher than expected. Our data suggest that, using this protocol, the GTP–QRET assay exhibits high accuracy within the standard cell count range typically employed in conventional experiments. However, as the cell size and content composition can vary between cell types, it is crucial to optimize the cell number and dilution factor for each cell line.

### Development of QT-Luc^GTP&ATP^ detecting cellular GTP and ATP down to 100 cells

The dual-detection method of GTP and ATP in a multi-well plate format has a high potential to provide a new technological platform applicable for many fields including, life, analytical, and food science as well as agriculture. Having validated the GTP-QRET applicability for cellular GTP detection, we set out to explore if GTP-QRET can equip additional functions to measure both GTP and ATP from the same sample. Towards this end, we adapted a commercial luminescence-based ATP assay from ATT Bioquest, since this ATP assay performed with only a single component addition is expected to work in a similar cell number (up to ~ 5000 cells/well^384^) to GTP-QRET. While GTP-QRET and ATP assay use luminescence-based detection, the readout is very different. GTP-QRET is monitored utilizing a TRL-signal readout using 620-nm and 340-nm excitation wavelengths (800 µs delay and 400 µs decay). On the other hand, in ATP assay, the total luminescence is monitored, and thus, no interference was expected when GTP detection was performed prior to the ATP measurement. We individually optimized the use of the ATP assay to enable simultaneous detection with GTP. For this, the added amount of ATP detection reagent was lowered from 25 to 10 µL, without any effect on the ATP detection in the concentration area of interest (data not shown). Under this condition, ATP detection also had a good MeOH tolerability and had no cross-reactivity to other nucleotides than ATP (see Electronic Supplementary Material Fig. S2 and S3).

As GTP-QRET and ATP detection methods showed the expected functionality individually, next, we combined the assays by detecting GTP and ATP from the same well. We selected a three-step protocol for the detection: addition of (1) nucleotide sample, (2) detection solution and TRL-signal monitoring, and (3) ATP detection solution and luminescence readout (see Electronic Supplementary Material Fig. S4). The first dual-readout tests were performed by adding GTP and ATP individually or together in Milli-Q water or GTP assay buffer. Based on these results, no change in GTP and ATP detection was seen in either case, when different addition were compared to each other (see Electronic Supplementary Material Fig. S5). For the GTP-QRET detection, the linear range in the presence of GTP with or without ATP was from 10 to 1000 nM, as ATP alone was not detected at these concentrations. Also, in the luciferase-based ATP assay, linear range was up to 1000 nM ATP without any GTP-related interferences (see Electronic Supplementary Material Fig. S5). The optimization showed good functionality using the dual-parametric single-well assay platform, and the obtained sensitivity is expected to be sufficient for GTP and ATP detection from cells. We named this new dual GTP and ATP detection system as QT-Luc^GTP&ATP^. The assay functionality was deemed satisfactory when using pure GTP and ATP solutions, as well as during the initial tests conducted with GTP-QRET alone. Good assay performance was observed when the cell number exceeded 100 cells per well in a 384-well plate, depending on the specific cell line and its nucleotide concentration (Fig. 1 and S5 see Electronic Supplementary Material).

**Fig. 2:**
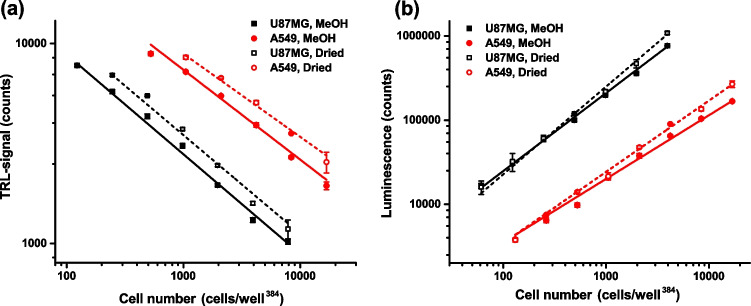
Linearity of the GTP and ATP concentration measurement from the cellular extract. Cell (U87MG, black, and A549, red) extract titration for GTP (**a**) and ATP (**b**) using MeOH solution samples (solid) and samples after MeOH evaporation and reconstitution in Milli-Q water (dashed). Data represents mean ± SD (*n* = 3)

For metabolomic analysis, MeOH-based extraction is a widely used method. However, protocol often requires MeOH evaporation and sample reconstitution in water before metabolomic analysis. Technically and ideally, the direct use of MeOH simplifies the protocol and will be the preferred option especially when multi-well plate cultures are used. To determine if QT-Luc^GTP&ATP^ is applicable for samples prepared by MeOH extraction, we used U87MG and A549 cell lines and two sample preparations, (1) direct dilution and detection from the 80% MeOH or (2) samples in Milli-Q water after MeOH evaporation. The cell titration analysis suggested that sample preparation by MeOH or reconstitution with water after MeOH extraction did not impact GTP or ATP detection (Fig. 2). In addition, both cell lines gave a linear response for GTP and ATP, with U87MG being detectable at lower cell numbers than A549. This is likely due to the higher nucleotide concentration in U87MG. Based on these results, the optimal cell number for the upcoming assays for U87MG is 200–2000 cells/well^384^ and 500–5000 cells/well^384^ in the case of A549. When these results were used to estimate the ratio between GTP and ATP, ATP concentration was shown to be five- to tenfold higher in comparison to GTP, A549 cells having the higher ratio. These results are in accordance with the ones reported previously [5]. Importantly, no similar interference with the extracts from higher cell number was detected as with GTP-QRET with 96-well cultures. Together, these results show that QT-Luc^GTP&ATP^ retains high assay functionality with samples prepared with the MeOH extraction.

### QT-Luc^GTP&ATP^ detects dynamic changes in cellular GTP concentrations

**Fig. 3:**
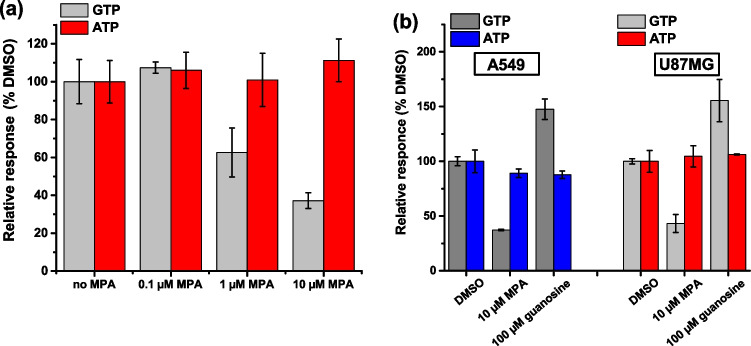
MPA and guanosine effect on GTP and ATP concentration in cellular extracts. **a** Concentration-dependent (0–10 µM) effect of MPA for GTP and ATP concentrations derived from U87MG cells, 2000 cells/well.^384^. **b** Single time-point and concentration effect of MPA (10 µM) with or without guanosine (100 µM) detected from U87MG and A549 cellular extract. Data represent mean ± SD (*n* = 3)

To assess the detection fidelity of QT-Luc^GTP&ATP^, we used pharmacological perturbation of cellular GTP levels using MPA, an inhibitor of the GTP-biosynthetic enzyme IMPDH [15–19]. Previous studies have shown that treatment of MPA decreases cellular GTP levels within 4 h, while ATP levels are transiently increased due to the reflux towards the ATP synthesis pathway. A previous study also showed significant growth suppression of U87MG cells by 10 µM MPA while the effect was moderate at 1 µM MPA [54]. U87MG cells (2000 cell/well^384^) were first treated with or without MPA for 8 h and subjected to QT-Luc^GTP&ATP^ assay. Consistent with the previous data detected by CE-MS by others [15], the QT-Luc^GTP&ATP^ assay shows that GTP concentrations were decreased approx. 50% and 30% to that of control (no MPA) by 1 µM and 10 µM MPA treatment, respectively (Fig. 3a). To further verify the functionality of QT-Luc^GTP&ATP^ assay, we next tested if the assay can detect the cellular GTP elevation induced by guanosine supplement, which increases cellular GTP concentration via HPRT1 in a time-dependent manner. For this, we also included A549 cells in addition to U87MG. Consistent with the Fig. 3a result, QT-Luc^GTP&ATP^ assay detected MPA-induced decrease in cellular GTP concentrations also in A549 cells, and importantly, QT-Luc^GTP&ATP^ assay detected the increase in cellular GTP concentration treatment with 100 µM guanosine (Fig. 3b). This is consistent with the previous CE–MS-based quantification [57]. Furthermore, we spiked a cell sample after lysis as a second control, and confirmed that a theoretically correct approximately twofold increase in GTP concentration was detected (data not shown).

### Detection fidelity of QT-Luc^GTP&ATP^ is comparable to CE-MS

**Fig. 4:**
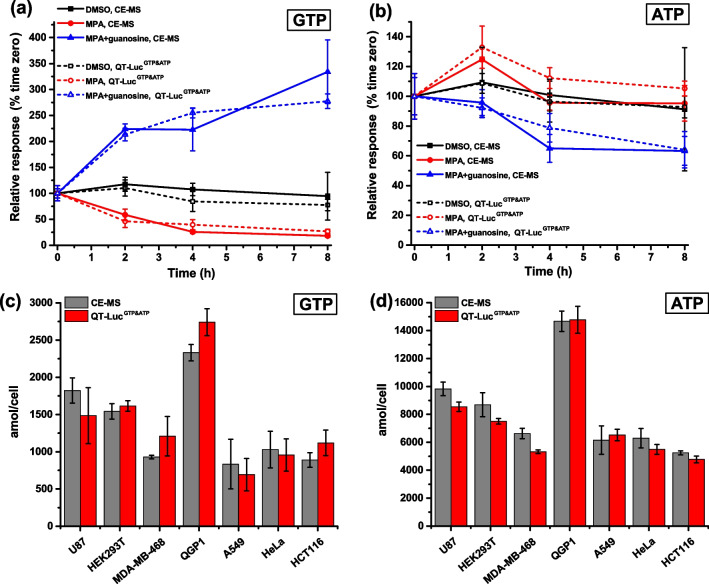
Comparison of the QT-Luc^GTP&ATP^ assay to the CE–MS reference method. Time-dependent effect of MPA (10 µM) with or without guanosine (100 µM) for GTP (**a**) and ATP (**b**) levels in U87MG cells detected using either QT-Luc^GTP&ATP^ or CE-MS. GTP (**c**) and ATP (**d**) levels in multiple different cell lines detected with QT-Luc.^GTP&ATP^ or CE-MS. Data represent mean ± SD (*n* = 3)

To further verify the accuracy of the QT-Luc^GTP&ATP^ method, we employed CE–MS-based quantification technology as a reference method [31, 58–61], First, we assayed the U87MG cells cultured in 6-well plates and modulated with MPA (10 µM) or guanosine (100 µM) (Fig. 4a). Cells designated for both assays were cultured and treated at the same time and manner before division for either assay (see Electronic Supplementary Material Fig. S6). In the presence of MPA, a near-maximal decrease in GTP concentration was achieved after 4 h of the treatment, while guanosine continued to elevate GTP levels even after 8 h. These trends were consistently observed with both GTP–QRET and CE–MS methods (Fig. 4a). As expected, ATP measurements displayed a less profound effect (Fig. 4b). We also determined CTP and UTP concentrations with CE-MS to estimate potential interferences for the GTP detection (see Electronic Supplementary Material Fig. S7a). In the case of untreated cells, GTP concentration was approximately 1800 amol/cell, whereas CTP and UTP concentrations were 1100 and 2200 amol/cell when monitored with CE-MS, respectively. Together, these data suggest that the QT-Luc^GTP&ATP^ method can detect cellular GTP concentration with accuracy comparable to CE-MS in U87MG cells, and without interferences occurring from nucleotide triphosphates or other cellular components.

To widen our analysis, we employed a diverse panel of cell lines, including SV40-transformed human embryonic kidney HEK293T, human lung adenocarcinoma A549, human breast adenocarcinoma MDA-MB-468, human pancreatic neuroendocrine cancer QGP1, human cervical adenocarcinoma HeLa, and human colorectal adenocarcinoma HTC116 cells. Across all cell lines examined, ATP concentrations were 5.3–7.4 (CE-MS) or 4.4–9.4 (QT-Luc^GTP&ATP^) times higher than to GTP. Despite the similar nucleotide ratios, greater variation was observed in their concentrations (Fig. 4). Notably, QGP1 cells had significantly higher GTP and ATP level compared to all other cell lines, with ATP and GTP concentrations approximately threefold higher than those in HCT116 for ATP and A549 for GTP, having the lowest concentrations.

From these cell lines, we also analyzed the CTP and UTP content using CE–MS (see Electronic Supplementary Material Fig. S7b). In all cell lines, CTP concentration (550–1400 amol/cell) was similar or lower in comparison to the GTP (830–2400 amol/cell) concentration. On the other hand, UTP concentrations (1500–4100 amol/cell) were slightly higher than those of GTP in all cell lines, which is typical for common cell lines [5]. In all cases, even the highest UTP concentrations are not anticipated to impact on GTP detection, as the 2A4^GTP^ Fab specificity against GTP is nearly 100-fold greater than for UTP (see Electronic Supplementary Material Fig. S2a). In addition, GDP interference is not expected, as its concentration was generally approx. 10 times lower in comparison to GTP, and in all cases, GDP was not even detected with CE-MS, falling below the detection limit (data not shown). Based on the CE–MS data, not only were the nucleotide triphosphate concentrations of interest changed from cell to cell and condition to condition, but also some other metabolites like NAD^+^ and UDP-glucose showed significant change in their concentration, indicating changes in the whole-cell metabolism (see Electronic Supplementary Material Fig. S8).

### QT-Luc^GTP&ATP^ increases the throughput of GTP and ATP detection

The commonly used nucleotide extraction protocol consists of multiple steps such as (1) cell washing and collection, (2) MeOH extraction, (3) centrifugation, (4) drying, and (5) dissolution to selected buffer [62]. We showed already with GTP-QRET that GTP is detectable from cellular extract from the 96-well plates, and that QT-Luc^GTP&ATP^ assay is not MeOH sensitive (Fig. 1 and S3 see Electronic Supplementary Material). However, as the dynamic range of the GTP-QRET and QT-Luc^GTP&ATP^ did not match when the nucleotide extraction was performed either in 96-well plates or higher-volume cultures, we decided to study QT-Luc^GTP&ATP^ further. U87MG and HEK293T cells were assayed, using 96-well plate cultures and 500 cells/well^384^ in the detection. Under these conditions, no interferences were detected, and calculated ATP and GTP concentrations correlated to those detected earlier from the cellular extracts (Fig. 4 and S9 see Electronic Supplementary Material). Five hundred cells/well^384^ were previously found optimal for GTP-QRET, and the observed interferences at higher cell number indicate that the protocol using extracts directly from the 96-well plate prefers a low cell number in the detection. This might be due to the high MeOH concentration, over the assay detection capacity (Fig. 2), caused by limited maximum cell capacity in the 96-well plates. Alternatively, centrifugation and sampling from the 96-well plate might be more prone to error. This needs to be considered if the nucleotide concentration of 500 cells/well^384^ is not sufficient, and it is expected to be fixed using alternative protocol in a 48-well plate. Overall, these results demonstrate the high-throughput potential of the QT-Luc^GTP&ATP^ method.

## Conclusions

The intrinsic roles of ATP and GTP in fueling cellular processes make them crucial in understanding disease mechanisms and discovering new therapeutic approaches. While existing technologies like HPLC and MS-based analysis have facilitated insightful investigations into cellular energy metabolism, their limitations in throughput, cost, and accessibility highlight the pressing need for alternative, high-throughput solutions. Recognizing this, we have developed and validated the novel GTP-QRET and QT-Luc^GTP&ATP^ technologies. By simultaneous monitoring of ATP and GTP levels in standard multi-well formats, we achieved results in a time- and cost-effective manner with a minimal number of cells. By surpassing the barriers presented by current methods, our GTP-QRET and QT-Luc^GTP&ATP^ platforms hold the potential to greatly enhance our understanding of the roles of nucleotides in cellular function and disease progression. Moreover, these methods are highly suited for drug screening, opening new avenues for therapeutic development, particularly for diseases linked with dysregulated GTP metabolism. However, despite our advancements, we recognize that the cell number, especially in the case of high-throughput 96-well plate cultures, is of high importance and in the current format, maximal cell number in high-throughput format is limited to approx. 500 cells/well^384^. Nonetheless, with the technological strides made in this study, we are one step closer to unraveling the complexities of cellular energy metabolism. We anticipate that our platforms will serve as the cornerstone for future studies in various fields, from medicine to food science, catalyzing the discovery of novel findings and advancements. While the full understanding of nucleotide functions remains a complex endeavor, our novel tools provide a significant stride forward, simplifying and expediting the journey toward this goal.

### Supplementary Information

Below is the link to the electronic supplementary material.

Supplementary file1 (DOCX 1.49 MB)
