# Supplementary material for: Homogeneous luminescent quantitation of cellular guanosine and adenosine triphosphates (GTP and ATP) using QT-LucGTP&ATP assay
Source: Anal Bioanal Chem. 2023 Sep 16;415(27):6689–700. doi: 10.1007/s00216-023-04944-9 (PMC10598090; doi:10.1007/s00216-023-04944-9)
Supplement: Supplementary file 1 — Supplementary file1 (DOCX 1.49 MB) [file 216_2023_4944_MOESM1_ESM.docx]

**Homogeneous luminescent quantitation of cellular guanosine and adenosine triphosphates (GTP and ATP) using QT-Luc^GTP&ATP^ assay**

**Kari Kopra,^1,*^ Randa Mahran,^1^ Titta Yli-Hollo,^1^ Sho Tabata,^2^ Emmiliisa Vuorinen,^1^ Yuki Fujii, ^3^ Iida Vuorinen,**^1^ **Aki Ogawa-Iio,^3^ Akiyoshi Hirayama,^2^ Tomoyoshi Soga,^2^ Atsuo T. Sasaki,^2,3,4,5^ and Harri Härmä^1,5^**

^1^ Department of Chemistry, University of Turku, Henrikinkatu 2, 20500 Turku, Finland.

^2^ Institute for Advanced Biosciences, Keio University, Tsuruoka, Yamagata 997-0052, Japan.

^3^ Department of Internal Medicine, University of Cincinnati College of Medicine, 3125 Eden Ave., Cincinnati, OH 45267-0508, USA.

^4^ Department of Clinical and Molecular Genetics, Hiroshima University Hospital, Hiroshima, 734-8551, Japan.

^5^ Co-senior authors

*Corresponding author: Kari Kopra, Email: [kari.kopra@utu.fi](mailto:kari.kopra@utu.fi), Phone: +358456339259

**Supplemental information**

**Table of contents**

**Supplemental figures**

**Fig. S1.** Chemical structure of GTP and other nucleotide triphosphates

**Fig. S2.** Nucleotide titration using luminescence-based detection system

**Fig. S3.** Methanol effect on GTP and ATP detection

**Fig. S4.** QT-Luc^GTP&ATP^ protocol for high throughput compatible GTP and ATP detection

**Fig. S5.** GTP and ATP detectability in different conditions

**Fig. S6.** Sample preparation for QT-Luc^GTP&ATP^ and CE-MS extracts

**Fig. S7.** CE-MS-based CTP and UTP detection from cells

**Fig. S8.** CE-MS-based UDP-Glucose and NAD^+^ detection from cells

**Fig. S9.** ATP and GTP detection from the 96-well plate extractions using QT-Luc^GTP&ATP^

**
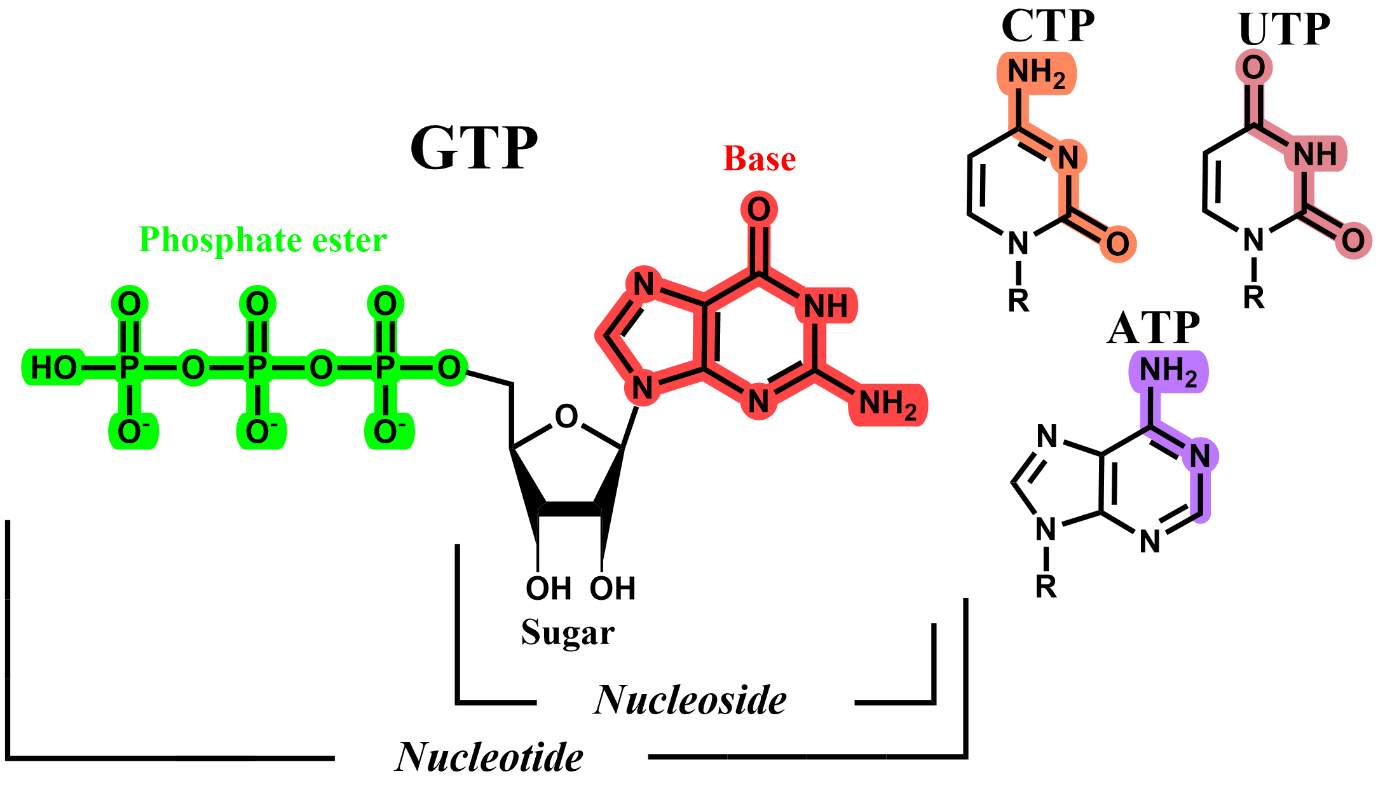
Fig S1.** Chemical structure of guanosine-5′-triphosphate (GTP) and other nucleotide triphosphates. All nucleotides consist of a varying base, a sugar moiety, and phosphate ester. These parts for GTP are shown in color, where the base is guanine (red), the sugar is β-D-ribose (black), and the triphosphate ester is highlighted in green. The base-and-sugar moiety is referred as a nucleoside, as together with the phosphate ester the molecule is called as a nucleotide. The base moiety separates GTP from other nucleotides. Purines, GTP and adenosine-5′-triphosphate (ATP) differ from atom position 6, as pyrimidines do not contain the second ring structure. Pyrimidines have different atoms in position 2 and 6. Bond lengths are not drawn in scale.

**Fig. S2.** Nucleotide titration using luminescence based detection systems. As the method is developed for cell-extracted nucleotides, all nucleotides and other metabolites are present during both GTP (A) and ATP (B) detections, which highlights the importance of high nucleotide specificity. In the used conditions, GTP was detected 33- and over 1300-times better than GDP and GMP, proving its triphosphate specificity. Similarly, GTP was detected 86-, 90- and over 300-times better than CTP, UTP, and ATP, proving its nucleoside specificity. In the case of ATP detection, none of the tested nucleotides gave detectable responses below 100 µM concentration, no longer useful for ATP detection. Data represent mean ± SD (n=3).

**Fig. S3.** Methanol (MeOH) effect on GTP and ATP detection. In the developed luminescent nucleotide detection, extracted nucleotides will be in MeOH containing solution, which impact on GTP (A) and ATP (B) detection was tested. MeOH concentration from 0 to 14% in GTP detection (10 µL) was tested, and as ATP is detected from the same wells (20 µL) the concentration corresponds 0-7% MeOH. In the case of GTP, both maximum and minimum TRL-signals increased slightly together with an increasing MeOH concentration. The increase in minimum TRL-signal was higher and thus the maximal S/B ratio in linear range was reduced from 6.4 to 4.9. In the case of ATP detection, effects were even smaller and no significant change either to S/B ratio or linearity was detected. As GTP detection responded sligtly to increased MeOH, a standard curve is important to be performed in the same MeOH concentration as in nucleotide extracts. Data represent mean ± SD (n=3).

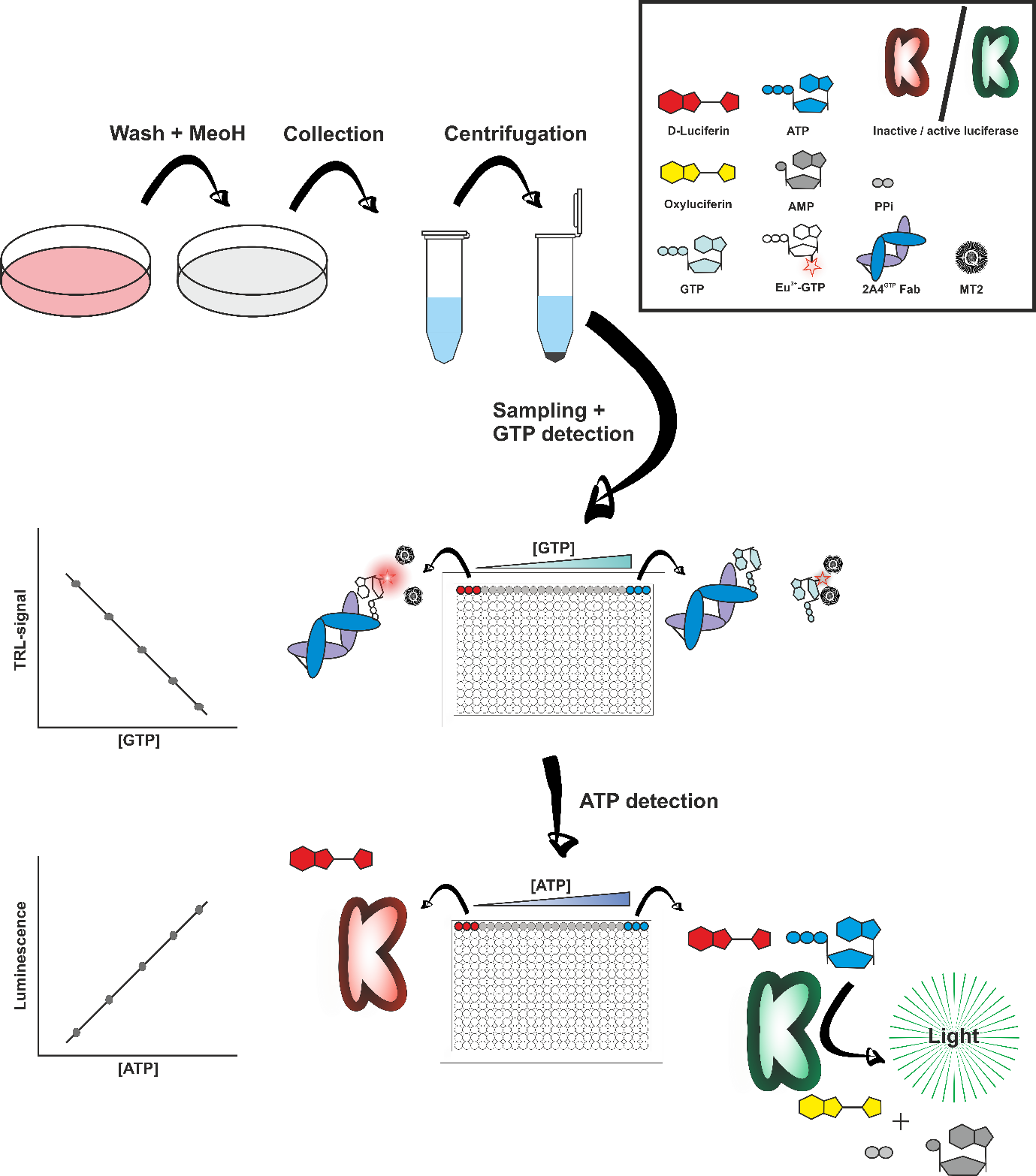


**Fig. S4.** QT-Luc^GTP&ATP^ protocol for high throughput compatible GTP and ATP detection. Cells for QT-Luc^GTP&ATP^ are cultured and treated either in plate or flask and extract is prepared using MeOH extraction. Cell extract and GTP/ATP standards are added in the plate prior to the GTP-QRET detection solution (10 nM Eu^3+^-GTP, 20 nM anti-GTP 2A4^GTP^ Fab, and 2.7 µM MT2). After 15 min incubation the TRL-signals were monitored (ex. 340 nm and em. 615 nm) and GTP concentration can be calculated based on the GTP standard. Thereafter, ATP detection solution was added in the same wells and luminescence signal was monitored after 15 min. Cellular ATP concentration can be calculated based on the ATP standard.

**Fig. S5.** GTP and ATP detectability in different conditions. Sampling induced effect on GTP (A) and ATP (B) detection using single GTP and ATP alone or together. No effect is observed when GTP and ATP are added to Milli-Q water or buffer. Similarly, no change in either GTP or ATP detectability is detected in the presence or absence of the non-specific nucleotide. Data represents mean ± SD (n=3).

**
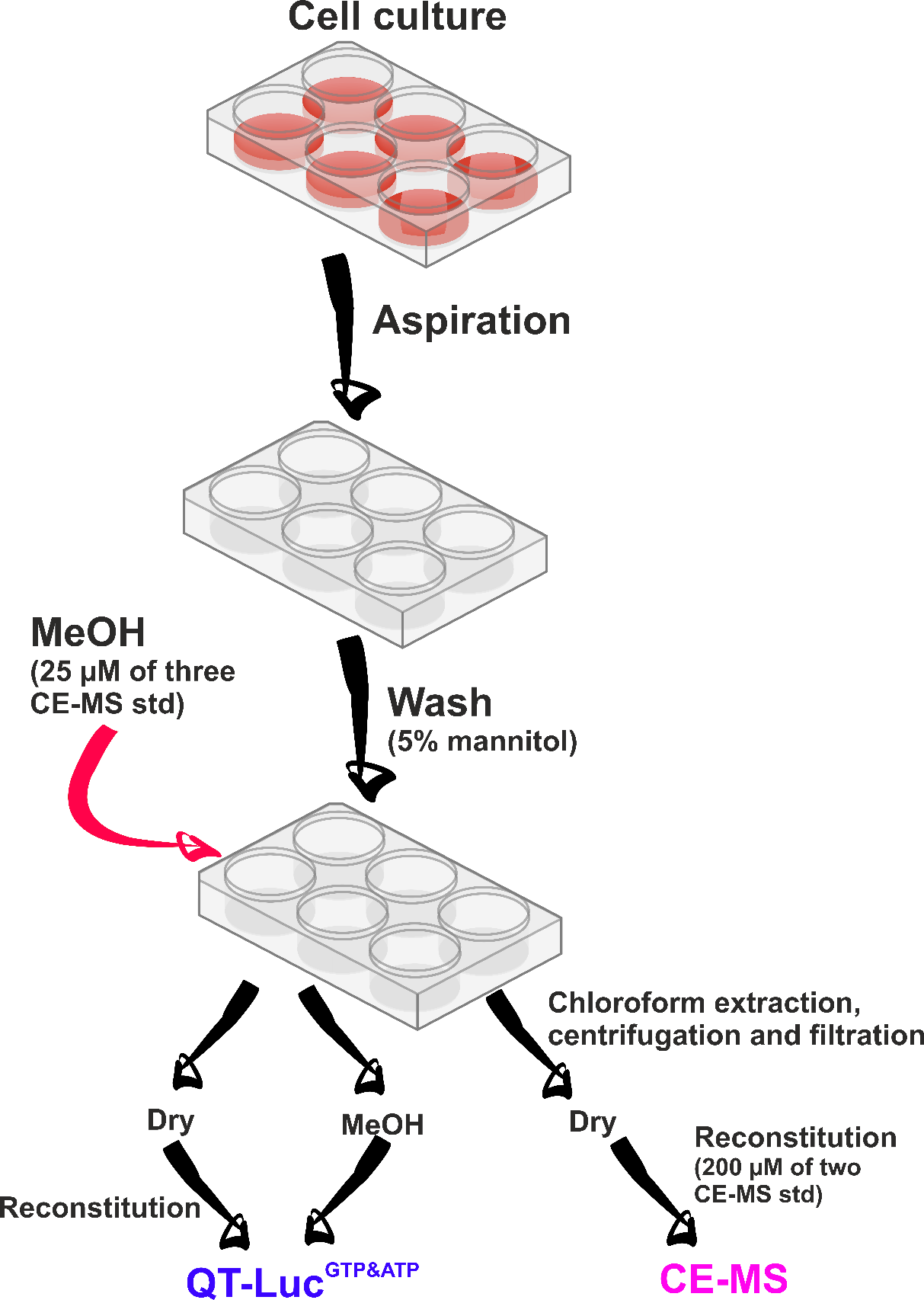
**

**Fig. S6.** Sample preparation for QT-Luc^GTP&ATP^ and CE-MS extracts. For a direct comparison of nucleotide concentration with two methods, samples were prepared similarly and divided before the final steps for CE-MS. Growth cells were first washed with 5% mannitol and thereafter disrupted with MeOH supplemented with CE-MS standards (methionine sulfone, ethane sulfonic acid, and d-camphor-10-sulfonic acid). Collected MeOH solution containing the nucleotides were then divided into three, two (dried or MeOH) samples for QT-Luc^GTP&ATP^ and one for CE-MS. The CE-MS samples were further processed to remove proteins (chloroform extraction, centrifugation, and filtration) and reconstituted in Milli-Q water with CE-MS standards (1,3,5-benzene tricarboxylic acid and 3-aminopyrrolidine).

**Fig. S7.** CE-MS-based CTP and UTP detection from cells. Capillary electrophoretic mass spectrometry (CE-MS) was mainly used as a control method for the developed QT-Luc^GTP&ATP^ detection system, but it can be used to monitor multiple molecules from the same samples. We monitored CTP and UTP concentrations from MPA ± guanosine treated U87MG cells (A) and from different non-treated cell lines (B). Interestingly, UTP concentration responded to MPA and guanosine treatment, but in an opposite fashion as the GTP concentration. After 8h MPA treatment, UTP concentration was 1.6-times over the baseline, as in the presence of guanosine the level was 1.7-times lower. UTP concentration also varied in different cell lines and it was highest in QGP1 and lowest in HCT116 cells. In either experiment, no significant change in CTP concentration was observed. Data represent mean ± SD (n=3).

**Fig. S8.** CE-MS-based UDP-Glucose and NAD+ detection from cells. We monitored UDP-Glu and NAD^+^ concentrations from MPA ± guanosine treated U87MG cells (A) and from different non-treated cell lines (B). UDP-Glu is a high-energy donor substrate and an important intermediate in several metabolic pathways and biosynthetic reactions. NAD^+^, on the other hand, links cellular metabolism to changes in signaling and transcriptional events. Thus both of these nucleotide related metabolite are directly linked to ATP and GTP and cell differentiation, it was expected that the concentration will vary at different conditions. Similar to UTP, UDP-Glu responded negatively to guanosine-induced increase in GTP concentration, as no change in NAD^+^ concentration was monitored. However, when cell lines were compared, NAD^+^ follows in most of the cases the UTP concentration. However, the UDP-Glu did not correlate with any of the more carefully analyzed metabolites. Data represent mean ± SD (n=3).

**Fig. S9.** ATP and GTP detection from the 96-well plate extractions using QT-Luc^GTP&ATP^. Cells cultured in a 96-well plate were monitored using 2-times 24 wells for the two cell lines (HEK293T and U87MG). Cells were cultured at the upper (solid) and lower (dashed) part of the plate, and plate centrifugation and MeOH treatment were performed in a plate. MeOH extracts (500 cells/well^384^) were monitored using QT-Luc^GTP&ATP^, using the same standards for all samples. Results showed no clear difference in the observed ATP/GTP ratio related to the 96-plate position, but a small difference in calculated ATP and GTP concentration was monitored. This might indicate difference in the absolute cell number, varying slightly from well to well, rather than variation in assay functionality. Using the ATP/GTP ratio, this small variation can be compensated, and the observed results are in line with those expected.
